# New Directions in Aesthetic Medicine: A Novel and Hybrid Filler Based on Hyaluronic Acid and Lactose Modified Chitosan

**DOI:** 10.3390/gels8050326

**Published:** 2022-05-23

**Authors:** Eva Daminato, Giulio Bianchini, Valerio Causin

**Affiliations:** 1 Dipartimento di Scienze Chimiche, Università degli Studi di Padova, Via Marzolo 1, 35131 Padova, Italy; eva.daminato@libero.it; 2 JoinTherapeutics Srl, Piazza del Popolo, 1, 22100 Como, Italy; giulio.bianchini@jointherapeutics.com

**Keywords:** hyaluronic acid, chitosan, crosslinking, rheology, dermal filler

## Abstract

Fillers based on crosslinked hyaluronic acid (HA) are becoming increasingly important in the field of aesthetic medicine, for example for treating wrinkles or for volumizing purposes. However, crosslinking agents are usually associated with toxicity and adverse reactions. The aim of this study is the development of an innovative technology to manufacture high performance HA-based fillers using minimal amounts of crosslinking agent. In this work, new fillers based on HA, functionalized with different amounts of 1,4-butanediol diglycidyl ether (BDDE) (degree of modification ranging between 3.5% and 8.8%) and formulated with a lactose modified chitosan (CTL), were investigated. The relative quantities of these polymeric building blocks in the formulations were 20–25 and 5 mg/mL for HA and CTL, respectively. Due to its cationic nature, CTL could interact with the anionic HA and enhance the elastic properties of the filler. Fillers manufactured with this novel technology (HACL-CTL) were characterized and compared with several fillers available in the market. In particular, resistance against hyaluronidase, swelling, cohesivity and rheological properties were investigated. Cohesivity, resistance to hydrolysis and swelling of HACL-CTL were comparable to commercial products. However, HACL-CTL fillers showed excellent elastic performance that reached 94% of elasticity in response to shear stresses. Surprisingly, these fillers also showed a resistance to compression higher than that of currently marketed products, making them very promising for their lifting effect.

## 1. Introduction

Hyaluronic acid (HA) is a linear polysaccharide abundant in skin and connective tissues composed of N-acetyl-D-glucosamine and D-glucuronic acid linked by alternating β-(1,4) and β-(1,3) glycosidic bonds. Hyaluronic acid-based dermal fillers are commonly used in aesthetic medicine because of their biocompatibility, biodegradability and non-immunogenicity [1]. The half-life of linear HA in the skin is about 12 h because of its rapid degradation by the enzyme hyaluronidase and by reactive oxygen species (ROS) [2,3]. To improve its resistance to hydrolysis, HA is cross-linked by different strategies [4], so that, once injected into the tissues, the filler can last for months or over a year [5]. The most widely used cross-linker is 1,4-butanediol diglycidyl ether (BDDE) [6,7] because of its safety at low concentrations. The epoxy groups of BDDE in alkaline conditions react preferentially with the primary hydroxyl group in the HA backbone, thus forming an ether bond [6].

The degree of cross-linking affects not only the stability, but also the rheological properties of the filler, like viscosity, viscoelasticity and cohesivity. Viscosity plays a key role during the injection of the filler because the lower the viscosity, the easier it is to extrude the gel through a needle. Furthermore viscosity, together with cohesivity, affects the tendency of the gel to remain at the injection site or to spread into the tissues. For these reasons, a dermal filler should preferably have high viscosity at low shear forces and low viscosity at high shear forces [8,9]. Viscoelastic properties are fundamental for dermal filler applications and aesthetic medicine specialists prefer to use different types of fillers for each area of the face, based on their characteristics: no product is suitable for all types of applications [10]. While the viscous modulus (G″) describes the viscous character, the elastic modulus G′ is related to the ability of the gel to recover its shape after a shear deformation; this elastic response to stresses is essential for applications in areas of the face that are particularly stressed by the facial muscles [11,12]. For example, fillers with low elastic modulus (G′) are suitable for the treatment of superficial wrinkles, while fillers with higher G′ are more suitable for the treatment of deeper lines and to obtain a volumizing effect [13].

In recent years, researchers have been trying to develop safe and stable high-performance fillers [10,14,15,16]. In this work, we analyze fillers (HACL-CTL) in which a lactose modified Chitosan, CHITLAC^®^ (CTL), is incorporated into the structure of cross-linked hyaluronic acid (HACL), in order to enhance the rheological properties of the gel. CTL is a chitosan derivative obtained through a reductive N-alkylation of primary amines by lactose moieties. In contrast to chitosan, it exhibits an improved solubility at neutral pH [17] and interesting biological properties [18,19,20,21]: for example, it induces aggregation of chondrocytes stimulating production of collagen and glycosaminoglycans [22]. CTL can be fully degraded by lysozyme [23] and partially by hyaluronidase [22], both enzymes commonly occurring in the human body. Due to its cationic nature, CTL in solution can interact with polyanions, such as alginate or hyaluronic acid, forming a polyelectrolyte complex [24,25,26,27,28]. A recent study [22] demonstrated that interactions between HA and CTL modify the chemico-physical properties of these polysaccharides: in particular a reduction of the charge and an increase of both elastic and viscous moduli were observed. This complex between HA and CTL has given promising results in the treatment of osteoarthritis, inducing a decrease in cartilage damage and synovial membrane inflammation. This treatment gave better results than the viscosupplementation of HA alone [29]. Moreover, the combination of HA and CTL showed many interesting biological effects, e.g., an attenuation of macrophage-induced inflammation, an inhibition of metalloproteinases expression and an antioxidant activity [30].

The scope of this work is to evaluate how the presence of CTL in the structure of an HA hydrogel affects the rheological performances of the gel. Resistance against hyaluronidase, swelling, cohesivity and rheological properties are investigated in order to assess the viability of these materials to use as dermal fillers, also by comparison with some commercially available products.

## 2. Results and Discussion

### 2.1. Development of HACL-CTL Technology

One of the aims of this study was to determine if the addition of Chitlac (CTL) could affect the properties of a cross-linked hyaluronic acid (HACL) and if a filler with outstanding performance could be obtained. With this in mind, first of all fillers based on the same HACL gel, with and without CTL, have been studied for comparison. The different samples were characterized by fixed CTL concentration, different concentration and molecular weight of HA used in the reticulation process and distinct degree of HA modification (MoD%) with BDDE. For the purpose of this study, HACL gels have been obtained from medium molecular weight hyaluronic acid (MMW) and high molecular weight hyaluronic acid (HMW). The synthesized samples are shown in Table 1 with the relative results of viscoelastic measurements.

Samples are coded as follows: molecular weight of HA—HACL—CTL—MoD.

Molecular weight of HA is indicated in the sample code as MMW or HMW. CTL appears in the name of the sample only if Chitlac is added to the formulation. As may be seen in Table 1, the quantity of HA in the formulation is always 20 mg/mL, except in sample MMW-HACL-CTL-7.0(25), where it is 25 mg/mL. CTL content is always, when this component is present, equal to 5 mg/mL.

Comparing samples MMW-HACL-3.5, MMW-HACL-7, MMW-HACL-CTL-3.5 and MMW-HACL-CTL-7.0, it is clear that the addition of CTL influences the mechanical behavior of the filler: in particular a statistically significant (t test *p*-value < 1 × 10^−5^) increase of the elastic modulus G′ and of the percentage of elasticity is observed with the same MoD%. This elasticizing effect is also observed from the value of the loss factor (tan δ), that is lower in samples containing CTL. Tan δ is expressed by the ratio G″/G′, therefore a decrease in its value reflects an improvement of the elastic behavior [13]. These data suggest that the electrostatic interactions between CTL and HA, due to their cationic and anionic nature respectively, allow us to obtain fillers with excellent viscoelastic performances and a structure that is more responsive to stresses.

Results of the rheological measurements of the samples MMW-HACL-CTL-3.5, MMW-HACL-CTL-7.0 and MMW-HACL-CTL-8.8 show that, by increasing the MoD% and keeping the polymer concentration constant, the elastic behavior improves. The value of G″ also increases, but not as much as G′; consequently, tan δ decreases and the elasticity percentage becomes higher. So, as expected, by increasing the cross-linking agent amount, the structure becomes more rigid and more effective in responding elastically to the stresses to which it is subjected. In particular, sample MMW-HACL-CTL-8.8 shows a surprising elasticity equal to 93%; however, such a highly cross-linked structure can be difficult to extrude with a syringe needle. Therefore, injectability and safety concerns suggest the choice of samples with lower MoD%.

HA concentration is another parameter that greatly affects the rheological properties: increasing the concentration of HA from 20 mg/mL (MMW-HACL-CTL-7.0) to 25 mg/mL (MMW-HACL-CTL-7.0(25)) produces a tripling of G′ and a two and half fold increase of G″; the percentage of elasticity consequently results higher. However, such a gel is difficult to inject, so it is not very suitable for medical purposes.

Finally, the influence of the molecular weight was evaluated. It was reported [16] that cross-linking of a high molecular weight HA is more effective and gives better results than cross-linking of a lower molecular weight polymer. It is therefore not surprising to see that sample HMW-HACL-CTL-7.0, made with a HMW HA, has a G′ of 374 Pa, which is much higher than the G′ of MMW-HACL-CTL-7.0 (141 Pa), made with MMW HA. However, while G′ increases by 165%, G″ increases by only 9%, resulting in an exceptional elasticity percentage of 94%.

### 2.2. Characterization of the Most Promising HACL-CTL Fillers

This preliminary study identified the most relevant preparation parameters that affect the rheological performances of HACL-CTL fillers. As shown in Table 2, fillers with a fixed concentration of 20 and 5 mg/mL for HA and CTL, respectively, and different MoD, were characterized and compared to commercial products (MKT). Together with rheological properties, resistance against hyaluronidase, swelling and cohesivity were investigated. Chosen commercial products can be divided into two subgroups characterized by two different brands and manufacturing technology: MKT1, MKT2 and MKT3 belong to the same group and share a production technology where reticulation with BDDE is performed on an HA matrix composed by two distinctive molecular weights [9], while MKT4, MKT5 and MKT6 belong to another market player and are manufactured according to the resilient hyaluronic acid (RHA) technology [10].

As shown in Table 2, sample HMW-HACL-CTL-3.8, although less cross-linked, has a better elasticity than sample MMW-HACL-CTL-5.7 due to the higher molecular weight of HA. The results of samples HMW-HACL-CTL-3.8, HMW-HACL-CTL-6.8 and HMW-HACL-CTL-7.0 show that by increasing the MoD%, G′ increases, while G″ decreases. As a result, a great increase is observed of the elastic percentage, which is always greater or equal than all the commercial samples examined (the difference is statistically significant between samples MKT2 and MKT4, with a *p*-value < 0.04, whereas it is statistically indistinguishable from the other market reference products). This can also be observed from the tan δ value, that goes from 0.22 for the less cross-linked sample to 0.06 for the most cross-linked one.

Results therefore highlight that, despite a lower HA content (20 vs 23 and 25 mg/mL), fillers produced with the HACL-CTL technology can reach or even overcome the elasticity values of commercial fillers. For instance, sample HMW-HACL-CTL-3.8, which is the one with the lowest value of elasticity obtained (82%), is as elastic as the soft gels MKT2 and MKT4 (*p*-value 0.57). Additionally, varying the BDDE amount and therefore the MoD%, viscoelastic properties can be modulated in order to satisfy all the needs for each type of application. For example, fillers with a high G′ like HMW-HACL-CTL-7.0 (374 Pa) could be used as a strong volumizer and for the treatment of deep wrinkles, while HMW-HACL-CTL-6.8 has a lower G′ value (232 Pa), but are still high enough to make it suitable for the treatment of wrinkles of lesser depth and for the volumization of particular areas, such as lips. Finally, HMW-HACL-CTL-3.8 could be an efficient soft gel for superficial treatments.

**Figure 1:**
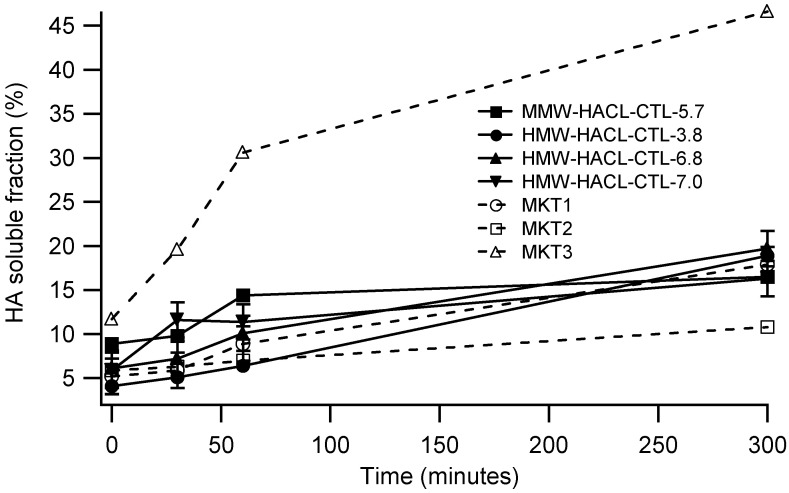
Hyaluronic acid (HA) soluble fraction after incubation with Bovine Testicular Hyaluronidase (BTH) 5 U/mL at 37 °C at different times (0, 30, 60 and 300 min). MKTx samples are commercial products analyzed for comparison. For clarity and to aid comparison, error bars are shown only for some of the samples. Lines are included only to guide the eyes.

For a more complete characterization, only MKT1, MKT2 and MKT3 were considered among the commercial materials, to highlight the differences between products manufactured with the same technology but with a different degree of crosslinking. Therefore, HACL-CTL fillers and MKT1, MKT2 and MKT3 have been subjected to enzymatic degradation tests that involve incubation with BTH 5 U/mL at 37 °C. Soluble fractions ranging from 50% to 80% after 5 h of incubation are commonly reported [10]. As shown in Figure 1, sample MKT2 seems to be the more resistant to hyaluronidase. Its initial soluble fraction is 5.9% and it doubles to 10.8% after 5 h. On the contrary, the degradation of MKT3 is extremely fast: after 30 min the soluble fraction is 19.6% and after 5 h it increases to 46.6%. HACL-CTL fillers have a very similar trend to MKT1 and have shown good resistance to degradation with a soluble fraction between 16.3% and 19.7% after 5 h. This highlights that HACL-CTL fillers, once implanted, have a longevity which is similar to that of fillers already introduced on the market. The filler degradability by hyaluronidase is important too, because overfilling or wrong placement of the gel can be corrected by administration of the enzyme, so a balance between degradability and stability is fundamental [10].

Swelling, i.e., the water uptake capacity of the gel, is inversely proportional to the crosslinking degree: a stronger cross-linked filler is able to absorb a lower quantity of solvent than a soft one. After injection, the filler for the first two months tends to swell, increasing the volume at the injection site [5]. Only later, the volume starts to decrease due to the bio-resorption process. It is therefore essential that the filler does not swell excessively to avoid aesthetic defects following the treatment. Swelling measurements of HACL-CTL fillers are reported in Table 3. Samples MMW-HACL-CTL-5.7, HMW-HACL-CTL-3.8 and HMW-HACL-CTL-6.8 have statistically indistinguishable values (*p*-value > 0.6), while the most cross-linked HMW-HACL-CTL-7.0 has the lowest swelling i.e., 89 (*p*-value < 0.00045). These values are comparable, or even lower, with those reported in the literature [9] for many commercial fillers. For instance, currently marketed products show swelling values ranging from 100 for highly cross-linked products to more than 300 for lightly cross-linked gels [9]. This confirms that HACL-CTL samples do not swell excessively, avoiding aesthetic defects that could follow the treatment [5].

**Figure 2:**
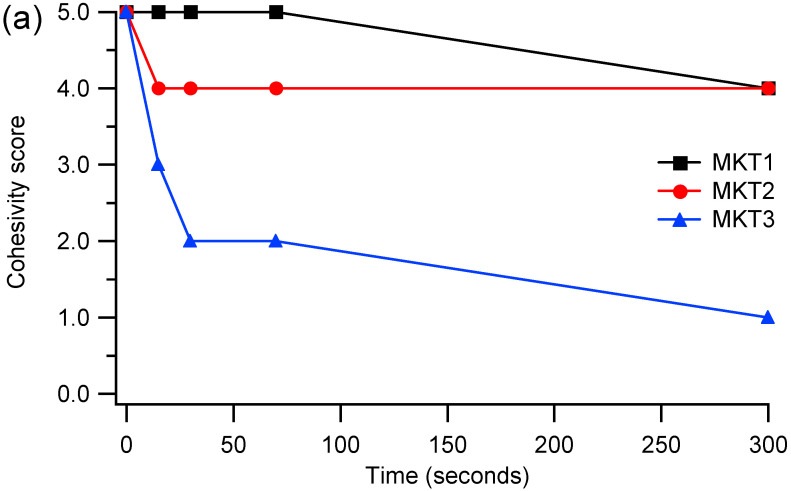

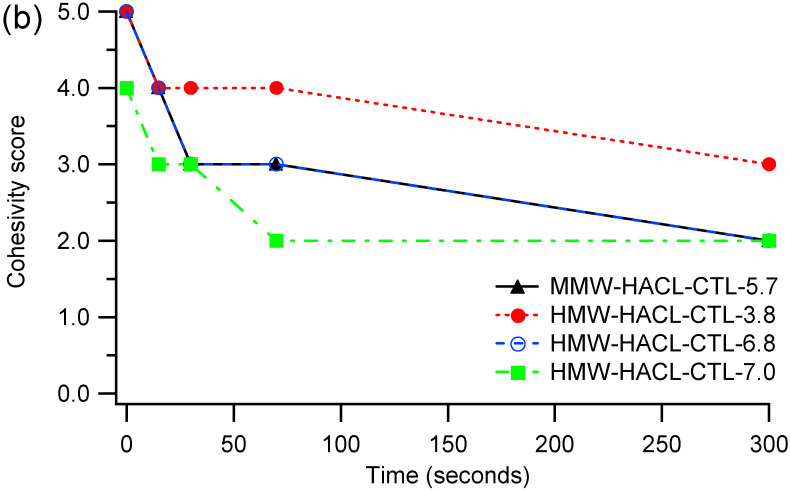
Cohesivity score in deionized water at 0, 30, 60 and 300 s of (**a**) commercial samples and (**b**) HACL-CTL fillers. Lines are added only to guide the eyes. Data regarding samples MMW-HACL-CTL-5.7 and HMW-HACL-CTL-6.8 are superimposed. Cohesivity scores are the following: 1: fully dispersed; 2: mostly dispersed; 3: partially dispersed, partially cohesive; 4: mostly cohesive; 5: fully cohesive.

Cohesivity describes the affinity between the gel molecules and is due to internal adhesion forces between HA units: non cohesive gels tend to dissociate [11]. Furthermore it describes the tendency to maintain an homogeneous distribution after injection and depends on HA concentration and crosslinking degree [10,11,12]. Cohesivity is usually expressed with a scale between 1 and 5, where 1 means fully dispersed and 5 means fully cohesive [31]. Cohesivity measurements (Figure 2) show that samples MKT1 and MKT2 have a good cohesivity, in particular MKT1 maintains a cohesivity score of 5, that drops to 4 after 5 min. On the contrary, MKT3 is the sample with the worst cohesivity, in fact it collapses to 2 after only 30 s. These data agree with what is reported in the literature [10,32,33]: there is an inverse correlation between G′ and cohesivity. The correlation seems to be the same for HACL-CTL fillers: HMW-HACL-CTL-3.8, with a G′ of 164 Pa, has a good cohesivity; HMW-HACL-CTL-6.8 with a G′ of 232 Pa has an intermediate cohesivity and HMW-HACL-CTL-7.0 with a G′ of 374 Pa is the least cohesive. Sample MMW-HACL-CTL-5.7, even if it has the lowest value of G′ (141 Pa), has an intermediate cohesivity: probably the difference in the trend is due to the lower molecular weight of HA, because a MMW HA was used instead of a HMW HA.

The normal force (F_N_) exerted by the gel when compressed is related to the capacity to resist vertical compression and to lift the tissues; it is reported [34] that F_N_ tends to increase with the volumizing capacity of the filler. Effectively, as shown in Table 4, the normal force exerted by HACL-CTL fillers increases with G′, which is a common parameter used to predict the mechanical properties of the gel: high values of G′ are correlated with a strongly cross-linked gel, suitable to deep implantation and with a good volumizing capacity. This trend can also be observed for samples MKT1, MKT2 and MKT3, that are produced with the same manufacturing technology. Instead, for samples MKT4, MKT5 and MKT6, made with another technology, there is a deviation from the trend because MKT5 has a higher value than MKT6. Maybe this could be due to other parameters, like the cohesivity of the gel, that can affect the measurement of the compression behavior [34]. In any case, these results show that the value of F_N_ depends on the type of manufacturing technology and highlight that HACL-CTL fillers exert a normal force higher than marketed products, suggesting a better lifting capacity. Indeed, sample MMW-HACL-CTL-5.7, among all the HACL-CTL fillers, has the lowest F_N_ value (1.90 N), but still higher than all the examined commercial samples (t-test always showed a statistically significant difference with a *p*-value < 0.0008). It is also described how HA-based fillers, after implantation, stimulate the production of collagen by fibroblasts due to an elongation in the morphology of the cells [35,36,37]. This phenomenon is related to the mechanical forces applied by the filler and could be predicted by the value of the normal force exerted by the gel [34]. Therefore, in addition to a greater lifting effect on the tissues, the HACL-CTL fillers could also favor the production of extracellular matrix components by activating the fibroblasts. However, cell cultures, biocompatibility and in vitro tests would be needed to confirm this latter hypothesis.

## 3. Conclusions

Fillers based on crosslinked hyaluronic acid are becoming increasingly important in the field of aesthetic medicine. In this work, we studied a novel manufacturing technology (HACL-CTL) to obtain fillers with improved rheological performance, exploiting the electrostatic interactions between a lactose modified chitosan and cross-linked hyaluronic acid. In particular, HACL-CTL fillers showed an excellent elasticity, displaying a dynamic structure able to respond effectively to stresses. By varying the degree of modification with BDDE, it is possible to modulate the properties of the filler obtaining both soft and hard gels, suitable for different applications. Finally, HACL-CTL fillers exert values of normal force much higher than competitors, suggesting a surprising ability to lift the tissues.

## 4. Materials and Methods

### 4.1. Materials

Lactose modified Chitosan, Chitlac^®^ and hyaluronic acid were kindly provided by Jointherapeutics. Chitlac^®^ has a molecular weight and a substitution degree close to 1 MDa and 60%, respectively. Medium molecular weight hyaluronic acid (MMW) ranges from 800 to 1500 kDa, while high molecular weight hyaluronic acid (HMW) exceeds 2000 kDa. BDDE was purchased from Tokyo Chemical Industry (Tokyo, Japan). PPI water was purchased from S.A.L.F. Spa (Bergamo, Italy). Commercial fillers (MKT) were purchased from two different leader of the market, so the groups MKT1-3 and MKT4-6 were produced with two different manufacturing technology: MKT1, MKT2 and MKT3 belong to the same group and shares a production technology where the reticulation with BDDE is performed on a HA matrix composed by two distinctive molecular weights [9], while MKT4, MKT5 and MKT6 belong to another market player and to the resilient hyaluronic acid (RHA) technology [10].

### 4.2. Preparation of HACL and HACL-CTL Fillers

HACL and HACL-CTL were provided by Jointherapeutics and were prepared according to the literature [38]. Briefly, HA was cross-linked with BDDE as described in the literature [2,16]. Then the bulk was buffered with phosphate saline buffer and a solution of Chitlac was added. Several samples were prepared varying the HA concentration (20 and 25 mg/mL), its molecular weight (MMW and HMW) and the amount of BDDE. After the synthesis, all the samples were steam sterilized in an autoclave with a 121 °C/15 min cycle.

### 4.3. Oscillatory Shear-Stress Test

The oscillatory test shear-stress test was performed with a rheometer (Kinexus lab+, Malvern Instruments, Malvern, UK) equipped with a 2 cm parallel plates geometry (gap 0.5 mm). Measurements were made at 20 °C in frequency sweep mode in the range 0.01–10 Hz by applying a shear stress of 5 Pa within the linear viscoelastic region (LVER). Three replicates were performed on each sample. The LVER was identified in amplitude sweep stress controlled mode with a frequency of 1 Hz and varying the shear stress from 0.1 to 10,000 Pa.

The percentage of elasticity (*E*%) of the filler is calculated with the following formula [13]:

(1) $$E\%=\frac{G^{\prime }}{G^{\prime }+G^{\prime \prime }}\times 100,$$

where G′ is the elastic modulus and G″ is the viscous modulus. The loss factor (tan δ) is the ratio G″/G′ and indicates whether the gel is more elastic or more viscous: if tan δ < 1 elastic behavior prevails [11].

### 4.4. Enzymatic Hydrolysis

The filler was incubated in the presence of a bovine testicular hyaluronidase (H3884) solution (BTH 5 U/mL) at 37 °C with no stirring. At different incubation time (0, 30 min, 1 h, 5 h) the sample was filtered on a PTFE membrane filter with a 0.45 µm pore size and the aqueous phase was recovered and diluted to determine the HA content by carbazole assay [39]. Then, 1 mL of the solution was mixed with 5 mL of disodium tetraborate 0.025 M in sulphuric acid (96%) and was heated at 98 °C for 10 min. Then, 0.2 mL of a 0.125% (*w*/*v*) carbazole solution in ethanol were added and the solution was heated at 98 °C for 15 min obtaining a pink/purple solution. The absorbance was measured at 530 nm by UV-Vis spectroscopy (Cary 100 UV-Vis) and the HA soluble fraction was calculated as:

(2) $$Soluble\;fraction\;\left(\%\right)=\frac{\mathrm{HA}\;concentration\;in\;the\;permeate\;\left(\frac{\mathrm{mg}}{\mathrm{mL}}\right)}{total\;\mathrm{HA}\;concentration\;\left(\frac{\mathrm{mg}}{\mathrm{mL}}\right)}\cdot 100.$$

The enzymatic degradation was monitored following the increase in the soluble fraction during the incubation time.

Uncertainty in soluble fraction values were assessed by repeating the hydrolysis tests of selected samples (MKT2, HMW-HACL-CTL-6.8 nd HMW-HACL-CTL-7.0) 3 times and was found to always be less than ±2%. This was taken as the uncertainty on all remaining samples.

### 4.5. Swelling

Of each sample, 1g was weighted and then incubated in 10 mL of phosphate buffer solution at 37 °C. When the equilibrium swelling was reached, the solvent in excess was removed and the gel was weighted. The swelling degree [9] was calculated as:

(3) $$Swelling\;\left(\frac{g}{g}\right)=\frac{Hydrated\;sample\;mass\;\left(g\right)}{Dry\;sample\;mass\;\left(g\right)}.$$

Three replicate measurements were performed for each sample.

### 4.6. Cohesivity

Cohesivity depends on the affinity between gel molecules and represents the ability of the filler to not dissociate and maintain an homogeneous distribution after injection [8]. The filler was mixed with a colorant and loaded into a syringe, then it was extruded into a 600 mL beaker containing deionized water under stirring. The cohesivity was evaluated at 0, 15, 30, 70 and 300 s by using the five-point visual Gavard–Sundaram Cohesivity Scale [31]: 1: fully dispersed; 2: mostly dispersed; 3: partially dispersed, partially cohesive; 4: mostly cohesive; 5: fully cohesive.

### 4.7. Normal Force Measurement

Measurements were carried out with a rheometer (Kinexus lab+, Malvern Instruments) equipped with a 2 cm parallel plates geometry. The normal force was measured at rest for a gap between the parallel plates of 0.15 mm. Three replicates were taken for each sample.

### 4.8. Degree of Modification (MoD%)

Under alkaline conditions, the epoxy groups of BDDE react with the hydroxyls of HA to form derivatives of 1,4-dibutanediol dipropan-2,3-diolyl ether (BDPE). These can bind to HA with both ends, allowing the effective cross-linking of polymeric chains, or only with one end, forming a sort of pendant. The degree of modification (MoD%) is the stoichiometric ratio between the sum of mono- and double-linked BDPE residues and HA disaccharide units [10]. The degree of modification was determined by ^1^H-NMR spectroscopy (Bruker 200 MHz) after acid hydrolysis. In particular, the samples were diluted to 4 mg/mL of HA with HCl 0.1 M and were kept under stirring at 75 °C overnight. The samples were then cooled to room temperature, neutralized with NaOH 0.25 M and precipitated with isopropanol. The precipitate was isolated, dried in an oven at 45 °C and dissolved in D_2_O for ^1^H-NMR analysis. The degree of modification (*MoD*%) was calculated from the integrals of the signals at 1.5 and 1.9 ppm using the following formula:

(4) $$MoD\left(\%\right)=\frac{I\left(1.5\;\mathrm{ppm}\right)/4}{I\left(1.9\;\mathrm{ppm}\right)/3}\cdot 100.$$

### 4.9. Statistical Tests

In order to assess the statistical significance of comparisons between the data regarding different samples, 2 sample t-tests, with pooled variance, using a 2-tailed distribution, were applied at a 95% confidence level.

## Figures and Tables

**Table 1:** Synthetic parameters and viscoelastic results of several samples. * Viscoelastic measurements were performed at a 1 Hz.

Sample	HA (mg/mL)	CTL (mg/mL)	MoD% (%)	Mw HA (kDa)	G′ (Pa)	G″ (Pa)	Elasticity (%)	tan δ
MMW-HACL-3.5	20	0	3.5	800–1500	27 ± 1	15 ± 1	64 ± 4	0.56 ± 0.04
MMW-HACL-7	20	0	7.0	800–1500	91 ± 4	16 ± 1	85 ± 6	0.18 ± 0.01
MMW-HACL-CTL-3.5	20	5	3.5	800–1500	35 ± 2	18 ± 1	66 ± 4	0.51 ± 0.04
MMW-HACL-CTL-7.0	20	5	7.0	800–1500	141 ± 7	22 ± 1	86 ± 6	0.16 ± 0.01
MMW-HACL-CTL-8.8	20	5	8.8	800–1500	643 ± 32	45 ± 2	93 ± 6	0.07 ± 0.01
MMW-HACL-CTL-7.0(25)	25	5	7.0	800–1500	425 ± 21	54 ± 3	89 ± 6	0.13 ± 0.01
HMW-HACL-CTL-7.0	20	5	7.0	>2000	374 ± 19	24 ± 1	94 ± 6	0.06 ± 0.01

* HA: hyaluronic acid concentration; CTL: Chitlac concentration; MoD: degree of modification as defined in par. 4.8; Mw HA: molecular weight of hyaluronic acid; G′: elastic modulus; G″: viscous modulus; tan δ: loss factor.

**Table 2:** Viscoelastic properties at 1 Hz of selected samples produced in this work and of some commercial samples (MKT 1–6). * Sample MMW-HACL-CTL-5.7 was prepared with medium molecular weight hyaluronic acid, while HMW-HACL-CTL-3.8, HMW-HACL-CTL-6.8 and HMW-HACL-CTL-7.0 with high molecular weight hyaluronic acid.

Sample	HA (mg/mL)	CTL (mg/mL)	MoD% (%)	G′ (Pa)	G″ (Pa)	Elasticity (%)	tan δ
MMW-HACL-CTL-5.7	20	5	5.7	141 ± 7	22 ± 1	86 ± 6	0.16 ± 0.01
HMW-HACL-CTL-3.8	20	5	3.8	164 ± 8	37 ± 2	82 ± 6	0.22 ± 0.02
HMW-HACL-CTL-6.8	20	5	6.8	232 ± 11	34 ± 2	87 ± 6	0.15 ± 0.01
HMW-HACL-CTL-7.0	20	5	7.0	374 ± 19	24 ± 1	94 ± 6	0.06 ± 0.01
MKT1	25	0	17.7	179 ± 9	22 ± 1	89 ± 6	0.12 ± 0.01
MKT2	25	0	14.0	489 ± 24	133 ± 7	79 ± 6	0.27 ± 0.02
MKT3	25	0	22.2	625 ± 31	45 ± 2	93 ± 6	0.07 ± 0.01
MKT4	23	0	5.1	183 ± 9	55 ± 3	77 ± 5	0.30 ± 0.02
MKT5	23	0	6.0 [10]	232 ± 11	52 ± 3	82 ± 6	0.22 ± 0.02
MKT6	23	0	6.8 [10]	308 ± 15	40 ± 2	88 ± 6	0.13 ± 0.01

* HA: hyaluronic acid concentration; CTL: Chitlac concentration; MoD: degree of modification as defined in par. 4.8; G′: elastic modulus; G″: viscous modulus; tan δ: loss factor.

**Table 3:** Swelling results of HACL-CTL fillers in phosphate buffer solution at 37 °C.

Sample	Swelling
MMW-HACL-CTL-5.7	184 ± 19
HMW-HACL-CTL-3.8	177 ± 12
HMW-HACL-CTL-6.8	178 ± 15
HMW-HACL-CTL-7.0	89 ± 8

**Table 4:** Normal force (F_N_) exerted and elastic modulus (G′) at 1 Hz of HACL-CTL fillers and commercial products.

Sample	F_N_ (N)	G′ (Pa)
MMW-HACL-CTL-5.7	1.90 ± 0.05	141 ± 7
HMW-HACL-CTL-3.8	2.31 ± 0.07	164 ± 8
HMW-HACL-CTL-6.8	2.63 ± 0.13	232 ± 11
HMW-HACL-CTL-7.0	2.84 ± 0.03	374 ± 19
MKT1	0.77 ± 0.06	179 ± 9
MKT2	0.83 ± 0.03	489 ± 24
MKT3	1.53 ± 0.11	625 ± 31
MKT4	1.12 ± 0.08	183 ± 9
MKT5	1.62 ± 0.02	232 ± 11
MKT6	1.47 ± 0.27	308 ± 15

## Data Availability

The data that support the findings of this study are available from the corresponding author upon reasonable request.
